# Evaluation of efficacy and safety of sorafenib in kidney cancer patients aged 75 years and older: a propensity score-matched analysis

**DOI:** 10.1038/s41416-018-0129-3

**Published:** 2018-06-12

**Authors:** Katsunori Tatsugami, Mototsugu Oya, Koki Kabu, Hideyuki Akaza

**Affiliations:** 1 Department of Urology, Graduate School of Medical Science, Kyushu University, 3-1-1, Maidashi, Higashi-ku Fukuoka City, 812-8582 Japan; 2 Department of Urology, Keio University School of Medicine, 35 Shinanomachi, Shinjuku-ku, Tokyo 160-8582 Japan; 3 Medical Affairs KI Oncology, Bayer Yakuhin, Ltd., 1-6-5, Marunouchi, Chiyoda-ku, Tokyo 100-8265 Japan; 4 Department of Strategic Investigational on Comprehensive Cancer Network, Interfaculty Institute in Information Studies/Science and Technology, The University of Tokyo, 7-3-1, Hongo, Bunkyo-ku, Tokyo 113-8654 Japan

**Keywords:** Targeted therapies, Renal cell carcinoma

## Abstract

**Background:**

The average age of patients diagnosed with renal cell carcinoma (RCC) is increasing, but a limited number of reports have described therapy of tyrosine kinase inhibitor for elderly RCC patients. Hence, we analysed the efficacy and safety of sorafenib in elderly patients aged ≥75 years with advanced RCC.

**Methods:**

Data were extracted from <75-year-old and ≥75-year-old patient groups, matching those demographics considered to affect prognosis. Differences in patients’ characteristics, dose modification, adverse events, tumour response, progression-free survival, and renal function (glomerular filtration) were evaluated between the groups.

**Results:**

From 2536 and 703 patients aged <75 and ≥75 years, respectively, 397 pairs were matched. Median daily dose was higher and duration of treatment longer in patients <75 years; however, progression-free survival and tumour response were similar in both age groups. Incidence of all adverse events was not significantly different between groups. The proportion of patients discontinuing treatment was higher in patients ≥75 years, but there was no significant difference between groups in the number patients discontinuing due to adverse events.

**Conclusions:**

For patients aged ≥75 years, sorafenib treatment had minimal additional negative impact compared to younger patients and showed similar efficacy and safety without reducing renal function.

## Introduction

Renal cell carcinoma (RCC) is the third most common cancer among urological cancers, and accounts for 3% of all cancers in adults.^1^ The incidence of RCC peaks between ages 60 and 70 years.^2^ Patients with RCC aged > 65 years account for about 50% of those diagnosed in the USA and almost 70% of those dying from this tumour.^3,4^ It is anticipated that the incidence of RCC patients will account for more than 50% due to recent ageing society.

Since the development of molecular targeted agents, especially those that inhibit the vascular endothelial growth factor receptors (VEGFRs), a paradigm shift has occurred in the treatment of patients with RCC over the past decade, leading to improvements in the prognoses of these patients.

Elderly patients are a heterogeneous group in which there may be disparities between chronological age and biological age, and the natural history of the disease and response to the treatment may be different from those of younger patients. The International Society of Geriatric Oncology indicated that ‘when considering the most appropriate drug to use in a particular patient, the toxicity profiles of the individual targeted agents – and any implications for specific comorbid conditions – should be taken into account’.^5^ Specifically, for the treatment of elderly patients with cancer, the guidelines suggest the following should be considered: (i) any decrease in the functional capacity of the major organs and the impact of treatment due to complication; (ii) choice of therapeutic agents and any necessary dose reductions due to impaired renal or liver function; and (iii) the impact of social factors and any decrease in quality of life should be considered.

To investigate the impact of older age on the efficacy and safety of sorafenib in the treatment of advanced RCC, we retrospectively analysed the data from real-world use of sorafenib in patients aged ≥75 years.

## Methods

### Study population

As reported in our earlier publications,^6,7^ these data were derived from Japanese patients with histologically or cytologically confirmed unresectable or metastatic RCC who started sorafenib treatment between February 2008 and September 2009. On the basis of a requirement from the Pharmaceutical and Medical Devices Agency (PMDA), these real-world data were collected prospectively from more than 3200 patients and analysed retrospectively.

### Study design

In this study, the background factors which affect the prognosis of patients with RCC were matched using propensity scores between ≥75 and <75 years of age, and the patients’ demographics, dose modification, AEs, tumour response, progression-free survival (PFS), and renal function (estimated glomerular filtration rate (eGFR)) were evaluated between these two groups.

Patients who had the following baseline data were selected for propensity score matching: Eastern Cooperative Oncology Group performance status (ECOG PS), tumour, node, metastasis (TNM) classification, prior surgery, prior systemic therapy, tumour histology, metastases (liver, brain, bone, and others (including lymph node)), C-reactive protein (CRP), and 1999 Memorial Sloan Kettering Cancer Center (MSKCC) risk. A total 1589 patients aged ≥75 years and 397 patients aged <75 years were selected and were matched with each other, resulting in 397 matching pairs (794 patients). There were statistically significant differences in body weight and eGFR after matching because these two factors were excluded from matching due to possible physiological change by ageing.

The definition of observation period was the time from sorafenib treatment to the date of the final assessment of survival (if patient died, the date of death). The median observation periods (days (IQR)) before and after matching were 267 (286) and 266 (294), respectively.

### AEs

AE grades were summarised based on the Medical Dictionary for Regulatory Activities (MedDRA), version 15.0, and classified as serious or non-serious. Serious AEs included (a) hospital admission or extended admission, (b) permanent significant dysfunction/failure, (c) those that lead to congenital abnormalities, (d) other medically serious state, (e) life-threatening AEs, (f) death, (g) others that are medically regarded as important events or reactions.

### Statistical analysis

Student’s *t*-test or the Mann–Whitney *U* test were used for continuous variables, and the *χ*^2^ test was used for categorical data. PFS was calculated using the Kaplan–Meier method. SAS version 9.1 or higher (SAS Institute Inc., Cary, NC, USA) was used for all statistical analysis.

## Results

### Demographics in <75-year-old and ≥75-year-old patients

Before matching, each patient’s demographics, excluding primary disease and metastatic site, differed between both groups. After matching, many of these differences between <75-year-old and ≥75-year-old groups were removed, but not (mean ± SD) age (63.0 ± 8.5 years vs. 78.1 ± 2.6 years, *P* *<* 0.0001), body weight (59.6 ± 11.6 kg vs. 55.3 ± 9.8 kg, *P* *<* 0.0001), and eGFR (53.7 ± 19.5 mL/min/1.73 m^2^ vs. 47.5 ± 15.5 mL/min/1.73 m^2^, *P* *<* 0.0001), respectively (Table 1). The baseline co-morbidity of the patients was shown in appendix.

**Table 1:** Patient characteristics

	Before matching			After matching		
	<75 years	≥75 years	*P* value	<75 years	≥75 years	*P* value
Number of patients	2536	703		397	397	
Gender, *n* (%)						
Male	1939 (76.5)	502 (71.4)	0.0060	310 (78.1)	292 (73.6)	0.1357
Female	597 (23.5)	201 (28.6)		87 (21.9)	105 (26.5)	
Age, years	62.0 ± 9.1	78.4 ± 3.1	<0.0001	63.0 ± 8.5	78.1 ± 2.6	<0.0001
Weight, kg	60.1 ± 11.7	55.3 ± 9.8	<0.0001	59.6 ± 11.6	55.3 ± 9.8	<0.0001
BMI, kg/m^2^	22.5 ± 3.6	22.0 ± 3.1	0.0070	22.4 ± 3.5	22.0 ± 3.2	0.1712
ECOG PS, *n* (%)						
0	1682 (66.3)	401 (57.0)	<0.0001	227 (57.2)	225 (56.7)	0.8758
1	733 (28.9)	261 (37.1)		142 (35.8)	147 (37.0)	
≥2	121 (4.8)	41 (5.8)		28 (7.1)	25 (6.3)	
eGFR (mL/min/1.73 m^2^)	55.9 ± 22.0	47.9 ± 17.6	<0.0001	53.7 ± 19.5	47.5 ± 15.5	<0.0001
TNM stage, *n* (%)						
I	8 (0.3)	9 (1.3)	<0.0001	0 (0.0)	1 (0.3)	0.5297
II	5 (0.2)	4 (0.6)		0 (0.0)	1 (0.3)	
III	27 (1.1)	18 (2.6)		2 (0.5)	3 (0.8)	
IV	2492 (98.3)	672 (95.6)		395 (99.5)	392 (98.7)	
Unknown	4 (0.2)	0 (0.0)		0 (0.0)	0 (0.0)	
Prior surgery, *n* (%) yes/no	2139 (84.4) / 397 (15.7)	564 (80.2) / 139 (19.8)	0.0093	365 (91.9) / 32 (8.1)	367 (92.4) / 30 (7.6)	0.7914
Prior systemic anticancer therapy, *n* (%)						
Any	2034 (80.2)	538 (76.5)	0.0330	330 (83.1)	330 (83.1)	1.0000
No	502 (19.8)	165 (23.5)		67 (16.9)	67 (16.9)	
Primary disease^a^, *n* (%)						
Unresectable/metastatic RCC	2502 (98.7)	698 (99.3)	0.1757	397 (100.0)	397 (100.0)	1.0000
Other than unresectable/metastatic RCC	40 (1.6)	7 (1.0)	0.2539	0 (0.0)	0 (0.0)	1.0000
Subtype, *n* (%)						
Clear cell carcinoma	1867 (73.6)	497 (70.7)	0.3599	335 (84.4)	346 (87.2)	0.2638
Non-clear cell carcinoma	259 (10.2)	60 (8.5)		62 (15.6)	51 (12.9)	
Metastatic site, *n* (%)						
Any	2472 (97.5)	670 (95.3)	0.0028	392 (98.7)	387 (97.5)	0.1925
Bone	823 (32.5)	184 (26.2)	0.0015	106 (26.7)	115 (29.0)	0.4761
Brain	144 (5.7)	25 (3.6)	0.0252	19 (4.8)	17 (4.3)	0.7330
Liver	405 (16.0)	87 (12.4)	0.0188	48 (12.1)	48 (12.1)	1.0000
Lung	1809 (71.3)	491 (69.8)	0.4413	293 (73.8)	288 (72.5)	0.6888
Lung only	628 (24.8)	211 (30.0)		131 (33.0)	124 (31.2)	
Kidney	187 (7.4)	50 (7.1)	0.8138	27 (6.8)	32 (8.1)	0.4987
Other (including lymph nodes)	1115 (44.0)	287 (40.8)	0.1368	164 (41.3)	152 (38.3)	0.3843
AST (IU/L)	26.5 ± 31.1	24.9 ± 17.6	0.2353	25.5 ± 21.0	25.0 ± 18.5	0.7668
ALT (IU/L)	24.2 ± 28.5	20.0 ± 20.6	0.0006	23.8 ± 27.4	20.3 ± 23.0	0.0563
T-Bil (mg/dL)	0.55 ± 0.34	0.57 ± 0.61	0.4028	0.55 ± 0.32	0.57 ± 0.62	0.6742
LDH (mg/dL)	229.3 ± 260.1	225.9 ± 208.1	0.7711	207.5 ± 118.4	220.0 ± 228.5	0.3432
CRP, mg/dL	3.15 ± 5.20	2.53 ± 4.01	0.0122	2.19 ± 3.98	2.30 ± 3.98	0.7145
MSKCC risk (1999)^b^, *n* (%)						
Favourable	451 (17.8)	87 (12.4)	0.0066	81 (20.4)	70 (17.6)	0.1443
Intermediate	1413 (55.7)	414 (58.9)		265 (66.8)	259 (65.2)	
Poor	143 (5.6)	35 (5.0)		18 (4.5)	14 (3.5)	

Data are expressed as mean ± SD unless otherwise indicated.

*BMI* body mass index, *CRP* C-reactive protein, *ECOG PS* Eastern Cooperative Oncology Group performance status, *eGFR* estimated glomerular filtration rate, *MSKCC* Memorial Sloan Kettering Cancer Center, *RCC* renal cell carcinoma, *TNM* tumour, node, metastasis.

^a^Including multiple choices.

^b^Patients with any line of therapy

### Treatment with sorafenib

Comparing the <75-year-old and ≥75-year-old groups, there were significant differences in the median (IQR) starting dose (800 (0.0) mg vs. 800 (400.0) mg, *P* = 0.2702) and median (IQR) daily dose (538.5 (400.0) mg vs. 422.3 (458.2) mg, *P* < 0.0001), relative dose intensity (RDI) (69.4% vs. 61.8%, *P* < 0.001), and median (IQR) duration of treatment (7.6 (9.9) months vs. 5.6 (10.3) months) (Table 2). As for dose modification, the proportion of patients in whom the dose was reduced or interrupted was similar between the groups; however, the percentage of patients discontinuing treatment was significantly higher in the ≥75-year group (63.7% vs. 75.3%, *P* = 0.0015). AEs accounted for more than half the patients discontinuing sorafenib treatment in both groups; this proportion was higher in the ≥75-year group but this was not statistically significant (Table 2). In the <75-year group, the percentage of patients who discontinued due to insufficient efficacy was significantly higher than in the ≥75-year group (36.0% vs. 26.4%, *P* = 0.0155) (Table 2).

**Table 2:** Distribution of initial dose, median dose, dose modification, and reasons for treatment discontinuation

	<75 years (*N* = 397)	≥75 years (*N* = 397)	*P* value
Median starting dose, mg/day (IQR)	800 (0.0)	800 (400.0)	0.2702
Median daily dose, mg/day (IQR)	538.5 (400.0)	422.3 (458.2)	<0.0001
Relative dose intensity, %	69.4 ± 26.3	61.8 ± 28.0	<0.0001
Median duration of treatment, months (IQR)	7.6 (9.9)	5.6 (10.3)	0.0123
Dose modification, *n* (%)			
Reduction	222 (55.9)	238 (60.0)	0.2501
Interruption	164 (41.3)	186 (46.9)	0.1158
Discontinuation	253 (63.7)	299 (75.3)	0.0004
Reason for discontinuation, *n* (%)			
AEs	147 (58.1)	184 (61.5)	0.4117
Insufficient efficacy	91 (36.0)	79 (26.4)	0.0155
Others	29 (11.5)	44 (14.7)	0.2609

*AE* adverse event, *IQR* interquartile range

### Adverse events

The most common among all AEs in the <75-year group compared with the ≥75-year group were hypertension (33.3% vs 37.3%, respectively) and decreased appetite (6.6% vs 12.9%, respectively; *P* = 0.0027). Alopecia (18.9% vs 11.8%, respectively; *P* = 0.0059), hepatic dysfunction (17.4% vs 14.9%), and hypophosphataemia (9.6% vs 5.3%, respectively; *P* = 0.0214) were more common in the <75-year compared to the ≥75-year group, respectively (Table 3). As for serious AEs, there was no significant difference except for fever (0.8% vs 2.8%, respectively; *P* = 0.0310) and renal failure/renal dysfunction (0.5% vs 2.3%; *P*= 0.0336).

**Table 3:** Most common adverse reactions

Adverse event	All	Serious	<75 years (*N* = 397)		≥75 years (*N* = 397)		*P*-values^a^	*P*-values^b^
			All	Serious	All	Serious		
Any, *n* (%)	766 (96.5)	408 (51.4)	384 (96.7)	193 (48.6)	382 (96.2)	215 (54.2)	0.7004	0.1183
Hand and foot skin reaction, *n* (%)	463 (58.3)	48 (6.1)	241 (60.7)	26 (6.6)	222 (55.9)	22 (5.5)		
Hypertension, *n* (%)	280 (35.3)	18 (2.3)	132 (33.3)	7 (1.8)	148 (37.3)	11 (2.8)		
Rash, *n* (%)	211 (26.6)	61 (7.7)	116 (29.2)	29 (7.3)	95 (23.9)	32 (8.1)		
Lipase/ amylase increase, *n* (%)	197 (24.8)	5 (0.6)	100 (25.2)	1 (0.3)	97 (24.4)	4 (1.0)		
Diarrhoea, *n* (%)	170 (21.4)	15 (1.9)	93 (23.4)	4 (1.0)	77 (19.4)	11 (2.8)		
Alopecia, *n* (%)	122 (15.4)	0 (0.0)	75 (18.9)	0 (0.0)	47 (11.8)	0 (0.0)	0.0059	
Hepatic dysfunction, *n* (%)	128 (16.1)	44 (5.5)	69 (17.4)	24 (6.1)	59 (14.9)	20 (5.0)		
Cytopenia, *n* (%)	107 (13.5)	42 (5.3)	55 (13.9)	22 (5.5)	52 (13.1)	20 (5.0)		
Bleeding, *n* (%)	84 (10.6)	59 (7.4)	41 (10.3)	26 (6.6)	43 (10.8)	33 (8.3)		
Decreased appetite, *n* (%)	77 (9.7)	15 (1.9)	26 (6.6)	4 (1.0)	51 (12.9)	11 (2.8)	0.0027	
Mucositis, *n* (%)	65 (8.2)	5 (0.6)	30 (7.6)	5 (1.3)	35 (8.8)	0 (0.0)		
Hypophosphatemia, *n* (%)	59 (7.4)	0 (0.0)	38 (9.6)	0 (0.0)	21 (5.3)	0 (0.0)	0.0214	
Fatigue, *n* (%)	14 (1.8)	2 (0.3)	8 (2.0)	2 (0.5)	6 (1.5)	0 (0.0)		
Dysphonia, *n* (%)	52 (6.6)	0 (0.0)	20 (5.0)	0 (0.0)	32 (8.1)	0 (0.0)		
Fever, *n* (%)	45 (5.7)	14 (1.8)	17 (4.3)	3 (0.8)	28 (7.1)	11 (2.8)		0.0310
Renal failure/renal dysfunction, *n* (%)	23 (2.9)	11 (1.4)	6 (1.5)	2 (0.5)	17 (4.3)	9 (2.3)	0.0199	0.0336

^a^Pooled analysis of all AEs in <75 and ≥75 years old.

^b^Pooled analysis of serious AEs in <75 and ≥75 years old

### Tumour response

**Fig. 1:**
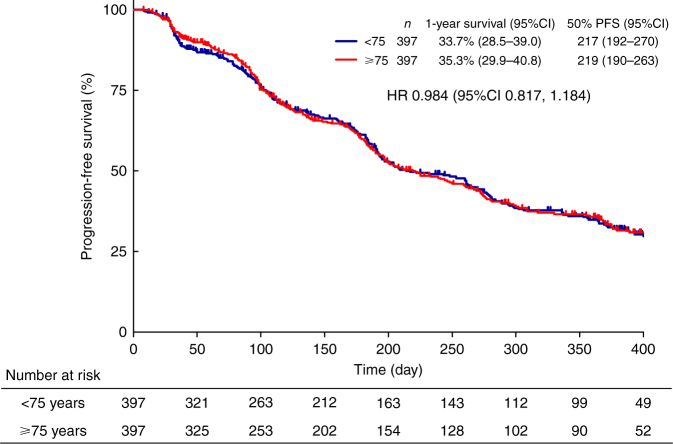
Progression-free survival. PFS progression-free survival, CI confidence interval, HR hazard ratio

Complete response (CR), partial response (PR), and stable disease (SD) rates in the <75-year and ≥75-year groups were 1.9% vs. 3.6%, 24.2% vs. 26.7%, and 60.2% vs. 56.4%, respectively. Objective response rate (CR + PR) and disease control rate (CR + PR + SD) in the <75-year and ≥75-year groups were 26.1% vs. 30.3% and 86.3% vs. 86.7%, respectively. Overall, sorafenib treatment provided a similar tumour response regardless of age (Table 4). As also shown in Fig. 1, the median PFS in the <75-year and ≥75-year groups was 217 days and 219 days, respectively, and the hazard ratio was 0.984 (95% confidence interval (CI): 0.817, 1.184) (Fig. 1).

**Table 4:** Tumour response to sorafenib

	All	<75 years (N = 397)	≥75 years (N = 397)	*P* value
CR, n (%)	20 (2.7)	7 (1.9)	13 (3.6)	0.3883
PR, n (%)	186 (25.4)	90 (24.2)	96 (26.7)	
SD, n (%)	427 (58.3)	224 (60.2)	203 (56.4)	
PD, n (%)	95 (13.0)	50 (13.4)	45 (12.5)	
NE, n (%)	4 (0.6)	1 (0.3)	3 (0.8)	
ORR, %	28.1	26.1	30.3	
DCR, %	86.5	86.3	86.7	

*CR* complete response, *DCR* disease control rate, *NE* not evaluable, *ORR* objective response rate, *PD* progressive disease, *PR* partial response, *SD* stable disease

### Influence on renal function

**Fig. 2:**
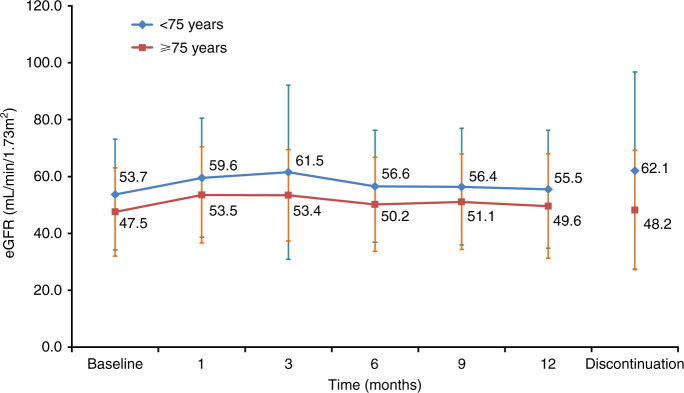
Change in renal function of overall patients over time according to age. eGFR estimated glomerular filtration rate

As the renal function of elderly patients may be significantly impaired before tyrosine kinase inhibitor (TKI) initiation and it has been reported that TKIs might reduce renal function, we investigated whether sorafenib treatment impacted the renal function of these elderly patients, and monitored renal function by measuring eGFR during the observational period. The baseline eGFR of <75-year and ≥75-year-old patients was 53.7 vs. 47.5 mL/min/1.73 m^2^, respectively, and this did not decrease over a 12-month period in either group (Fig. 2). When the eGFR of those who discontinued sorafenib treatment was analysed, it was not significantly changed from baseline (Fig. 2). This suggested that the maintained eGFR was not based on excluding low eGFR values of patients who dropped out of treatment.

## Discussion

A chronological age of 65 years or more is defined as ‘elderly’ or ‘older people’.^8^ Recently, the definition of elderly people has been reconsidered due to the ageing population in Japan, and a proposal from the Japan Gerontological Society and the Japan Geriatrics Society is that the definition be changed as follows: ‘pre-old age’ 65 to 74 years, ‘old age’ 75 to 90 years, and ‘super-old age’ (or ‘oldest-old age’) more than 90 years. There have been a limited number of reports that showed the safety and efficacy of a TKI in RCC patients aged ≥75 years. As for sorafenib, Procopio et al. performed comprehensive analysis of six clinical studies and two expanded access programs, and showed there was no difference in safety in patients aged between ≥75 and <75 years.^9^ However, in this report, patients’ demographic bias was not balanced by use of propensity score matching. Regarding another TKI, Miyake et al. reported the safety and efficacy of axitinib in ≥75-year and <75-year-old RCC patients,^10^ but these data were from a single centre, and only 28 patients were aged ≥75 years. Therefore, we compared real-world data on the safety and efficacy of sorafenib in patients aged <75 and ≥75 years using propensity score-matching analysis.

It is known that elderly patients with RCC often have comorbidities such as hypertension, cardiovascular disease and diabetes.^11^ Heng et al. reported that 35% of patients treated with molecular targeted agents in clinical practice did not meet the inclusion criteria of a general clinical study in terms of their comorbidities.^12^ Treatment efficacy such as tumour shrinkage, PFS, or overall survival in these patients was lower than that of elderly patients whose demographics were compatible with enrolment in a clinical study.^12^ For these reasons, the necessity for evaluation of the safety and the efficacy of molecular targeted agents in real-world clinical practice, with patients who might be excluded from clinical studies because of their comorbidities or complications becomes more important.

Although elderly patients are rarely excluded from the clinical studies of cancer because of their age, the numbers included may seem low because many were excluded due to comorbidities in their backgrounds. In the phase 3 TARGET study of sorafenib in patients with RCC, which had no age restrictions for enrolment, patients aged ≥65 years accounted for only 27.4% of all patients,^13^ which is lower than the age distribution of RCC previously reported.^14^ Before matching in this study, patients aged ≥75 and ≥65 years accounted for 21.7% (703/3239) and 56.9% (1842/3239), respectively (data not shown), which is higher than in the TARGET study,^13^ but lower than the age distribution of RCC (≥65 and ≥75 years: 69 and 40%, respectively).^14^ The reason for the difference in age distribution in this study in comparison to other reports is that some physicians were less familiar with the use of sorafenib, because it had only recently been approved. Hence, there was some physician bias that impacted the number of elderly patients enroled.

In this study, there were significant differences in body weight and renal function between the <75-year and ≥75-year groups before matching. To evaluate the impact of sorafenib on elderly patients with RCC, matching was conducted using the factors which were considered to affect the prognosis of these patients. Factors such as body weight and renal function which might change with age were excluded from matching because there would be a possibility to select specific population of elderly patients if these two factors were included in matching. Albiges et al. reported that the prognoses of metastatic RCC patients treated with targeted therapy were better in BMI ≥25 group than those of <25 group.^15^ Although the frequency of those with BMI ≥25 was 17.1% (553/3239) in patients of this study, there was no significant difference in BMI after matching.

The lower starting dose for elderly patients might reflect the higher numbers of those with low body weight and reduced renal function. The number of patients who discontinued treatment was higher in the ≥75-year group (*P* = 0.0004), but those who discontinued due to insufficient efficacy was lower in this group (*P* = 0.0155) (Table 2). In clinical practice, even when patients had similar AEs, older patients may be more likely to discontinue treatment by the physician’s decision or at their own request. Taking these situations into consideration, it might be difficult to continue the treatment of elderly patients while maintaining their quality of life and safety.

Generally, it is reported that there is a relationship between the anti-tumour effect of TKI and its RDI;^16–20^ however, in our study there was no significant difference in the anti-tumour effect in either group regardless of the lower RDI in the ≥75-year group. Although the reason for this discrepancy is unclear, the optimal drug concentration might be changed due to decrease in renal or liver function in elderly patients; therefore, there is a possibility that a lower than usual dose might achieve a sufficient anti-tumour effect in this patient population. The fact that the frequency of hypertension, a predictive factor of TKI treatment,^21–23^ was higher in ≥75-year-old patients reflected these physical status specific for elderly patients. As for the frequency of AEs during sorafenib treatment, hypertension and decrease in appetite were higher in the ≥75-year group, but there was no significant difference for other AEs (Table 3). This indicated that sorafenib treatment could be effective in elderly patients with lower doses due to metabolism differences.

It is known that the pharmacodynamics of sorafenib differ among individuals,^24^ and the serum concentration of sorafenib might have been retained to similar level between the groups because of the difference of metabolism in liver, possibly accounting for the lack of difference in AEs rates. Although it is necessary to elucidate the plasma concentration of sorafenib, the data were not collected in this regard.

It is considered that TKIs affect renal function because of their pharmacological mode of action. In the AXIS study comparing axitinib with sorafenib, an increase in serum creatinine of all grades was reported in 55 and 41% of patients, respectively.^25^ In the COMPARZ study comparing pazopanib with sunitinib, an increase in serum creatinine of all grades was reported in 32 and 46% patients, respectively.^26^ Since it is known that renal function may decrease in elderly patients, the impact of sorafenib on renal function was analysed. We found that the renal function remained stable in the ≥75-year-old patients over the duration of sorafenib treatment; there was no significant decrease in eGFR at treatment discontinuation, which indicated that renal impairment was not a factor in treatment discontinuation. Sorafenib treatment for RCC in elderly patients appears safe with respect to renal function.

One limitation of this study was that it was retrospective without randomisation. Propensity score matching apparently shows similar outcomes as a randomised study,^27^ but it cannot be denied that there were some biases: those for whom the data of matching factors were missing at baseline were excluded from the analysis. In addition, prognostic factors were used as matching factors; however, other factors such as lower mean starting dose or higher drug discontinuation rate were not considered for propensity score matching. Also, as mentioned earlier, because these data were obtained just after the approval of sorafenib, a healthcare professional bias could exist, as physicians without wide experience of sorafenib were possibly included in this study. In addition, this study was based entirely on data derived from a Japanese population, and therefore the results are not necessarily applicable to non-Japanese patients.

We evaluated the safety and efficacy of sorafenib in elderly patients with RCC by matching patients’ characteristics between two groups, aged <75 and ≥75 years, using propensity score matching. Sorafenib was well tolerated in the ≥75-year group, and efficacy, as measured by tumour response, was comparable to that in patients aged <75 years.

## Electronic supplementary material

Baseline co-morbidity

## Availability of data and material

The data that support the findings of this study are available from the corresponding author upon reasonable request.
