# Supplementary material for: Evaluation of efficacy and safety of sorafenib in kidney cancer patients aged 75 years and older: a propensity score-matched analysis
Source: Br J Cancer. 2018 Jun 12;119(2):241–7. doi: 10.1038/s41416-018-0129-3 (PMC6048135; doi:10.1038/s41416-018-0129-3)
Supplement: Supplementary file 1 — Baseline co-morbidity [file 41416_2018_129_MOESM1_ESM.docx]

Appendix

Baseline co-morbidity

| System organ class | Preffered terms | Before matching | |  | After matching | |
| --- | --- | --- | --- | --- | --- | --- |
|  |  | <75 year old (N=2536) | ≥75 year old (N=703) |  | <75 year old (N=397) | ≥75 year old (N=397) |
| Blood and lymphatic system disorders, n (%) |  | 6 (0.2) | 3 (0.4) |  | 1 (0.3) | 2 (0.5) |
|  | Anaemia | 2 (0.1) | 0 |  | 1 (0.3) | 0 |
|  | Disseminated intravascular coagulation | 0 | 1 (0.1) |  | 0 | 1 (0.3) |
|  | Iron deficiency anaemia | 1 (0.0) | 0 |  | 0 | 0 |
|  | Normochromic normocytic anaemia | 1 (0.0) | 0 |  | 0 | 0 |
|  | Polycythaemia | 1 (0.0) | 0 |  | 0 | 0 |
|  | Thrombocytopenia | 0 | 1 (0.1) |  | 0 | 0 |
|  | Nephrogenic anaemia | 1 (0.0) | 0 |  | 0 | 0 |
|  | Thymus enlargement | 0 | 1 (0.1) |  | 0 | 1 (0.3) |
| Cardiac disorders, n (%) |  | 105 (4.1) | 60 (8.5) |  | 23 (5.8) | 29 (7.3) |
|  | Acute myocardial infarction | 1 (0.0) | 1 (0.1) |  | 0 | 0 |
|  | Angina pectoris | 28 (1.1) | 14 (2.0) |  | 7 (1.8) | 8 (2.0) |
|  | Angina unstable | 1 (0.0) | 1 (0.1) |  | 0 | 0 |
|  | Aortic valve disease mixed | 1 (0.0) | 0 |  | 0 | 0 |
|  | Aortic valve stenosis | 1 (0.0) | 1 (0.1) |  | 0 | 1 (0.3) |
|  | Arrhythmia | 12 (0.5) | 6 (0.9) |  | 3 (0.8) | 4 (1.0) |
|  | Atrial fibrillation | 13 (0.5) | 9 (1.3) |  | 2 (0.5) | 5 (1.3) |
|  | Atrial flutter | 1 (0.0) | 0 |  | 0 | 0 |
|  | Atrioventricular block complete | 3 (0.1) | 1 (0.1) |  | 1 (0.3) | 1 (0.3) |
|  | Bradycardia | 1 (0.0) | 0 |  | 0 | 0 |
|  | Cardiac failure | 3 (0.1) | 5 (0.7) |  | 1 (0.3) | 2 (0.5) |
|  | Cardiac failure chronic | 1 (0.0) | 1 (0.1) |  | 0 | 0 |
|  | Cardiac failure congestive | 1 (0.0) | 0 |  | 0 | 0 |
|  | Cardiac hypertrophy | 2 (0.1) | 0 |  | 0 | 0 |
|  | Coronary artery stenosis | 1 (0.0) | 0 |  | 1 (0.3) | 0 |
|  | Mitral valve incompetence | 4 (0.2) | 0 |  | 0 | 0 |
|  | Mitral valve prolapse | 1 (0.0) | 0 |  | 1 (0.3) | 0 |
|  | Mitral valve stenosis | 0 | 1 (0.1) |  | 0 | 1 (0.3) |
|  | Myocardial infarction | 18 (0.7) | 8 (1.1) |  | 3 (0.8) | 4 (1.0) |
|  | Myocardial ischaemia | 2 (0.1) | 1 (0.1) |  | 0 | 0 |
|  | Prinzmetal angina | 1 (0.0) | 0 |  | 0 | 0 |
|  | Supraventricular tachycardia | 0 | 1 (0.1) |  | 0 | 0 |
|  | Ventricular extrasystoles | 0 | 2 (0.3) |  | 0 | 1 (0.3) |
|  | Ventricular tachycardia | 1 (0.0) | 0 |  | 0 | 0 |
|  | Atrial thrombosis | 1 (0.0) | 0 |  | 0 | 0 |
|  | Congestive cardiomyopathy | 1 (0.0) | 0 |  | 0 | 0 |
|  | Brugada syndrome | 1 (0.0) | 0 |  | 1 (0.3) | 0 |
|  | Cardiac valve disease | 1 (0.0) | 4 (0.6) |  | 0 | 1 (0.3) |
|  | Aortic valve disease | 1 (0.0) | 0 |  | 1 (0.3) | 0 |
|  | Stress cardiomyopathy | 1 (0.0) | 0 |  | 1 (0.3) | 0 |
|  | Sinus node dysfunction | 2 (0.1) | 4 (0.6) |  | 1 (0.3) | 1 (0.3) |
| Congenital, familial and genetic disorders, n (%) |  | 14 (0.6) | 0 |  | 2 (0.5) | 0 |
|  | Congenital cystic kidney disease | 1 (0.0) | 0 |  | 1 (0.3) | 0 |
|  | Diverticulitis Meckel's | 1 (0.0) | 0 |  | 0 | 0 |
|  | Double outlet right ventricle | 1 (0.0) | 0 |  | 0 | 0 |
|  | Fallot's tetralogy | 1 (0.0) | 0 |  | 0 | 0 |
|  | Haemangioma of retina | 1 (0.0) | 0 |  | 0 | 0 |
|  | Hepatic arteriovenous malformation | 1 (0.0) | 0 |  | 0 | 0 |
|  | Hydrocele | 1 (0.0) | 0 |  | 1 (0.3) | 0 |
|  | Hypertrophic cardiomyopathy | 1 (0.0) | 0 |  | 0 | 0 |
|  | Syringomyelia | 1 (0.0) | 0 |  | 0 | 0 |
|  | Ventricular septal defect | 1 (0.0) | 0 |  | 0 | 0 |
|  | Von Hippel-Lindau disease | 1 (0.0) | 0 |  | 0 | 0 |
|  | Adenomatous polyposis coli | 1 (0.0) | 0 |  | 0 | 0 |
|  | Atrioventricular septal defect | 1 (0.0) | 0 |  | 0 | 0 |
|  | Anomalous arrangement of pancreaticobiliary duct | 1 (0.0) | 0 |  | 0 | 0 |
| Ear and labyrinth disorders, n (%) |  | 8 (0.3) | 2 (0.3) |  | 0 | 2 (0.5) |
|  | Deafness | 3 (0.1) | 1 (0.1) |  | 0 | 1 (0.3) |
|  | Meniere's disease | 4 (0.2) | 0 |  | 0 | 0 |
|  | Sudden hearing loss | 1 (0.0) | 1 (0.1) |  | 0 | 1 (0.3) |
| Endocrine disorders, n (%) |  | 20 (0.8) | 12 (1.7) |  | 3 (0.8) | 8 (2.0) |
|  | Addison's disease | 0 | 1 (0.1) |  | 0 | 1 (0.3) |
|  | Adrenal insufficiency | 1 (0.0) | 1 (0.1) |  | 0 | 1 (0.3) |
|  | Basedow's disease | 2 (0.1) | 0 |  | 1 (0.3) | 0 |
|  | Cushing's syndrome | 0 | 1 (0.1) |  | 0 | 1 (0.3) |
|  | Goitre | 5 (0.2) | 1 (0.1) |  | 1 (0.3) | 1 (0.3) |
|  | Hyperthyroidism | 5 (0.2) | 4 (0.6) |  | 1 (0.3) | 1 (0.3) |
|  | Hypothyroidism | 2 (0.1) | 3 (0.4) |  | 0 | 2 (0.5) |
|  | Thyroid disorder | 2 (0.1) | 0 |  | 0 | 0 |
|  | Thyroiditis chronic | 1 (0.0) | 0 |  | 0 | 0 |
|  | Autoimmune thyroiditis | 2 (0.1) | 1 (0.1) |  | 0 | 1 (0.3) |
| Eye disorders, n (%) |  | 28 (1.1) | 20 (2.8) |  | 3 (0.8) | 8 (2.0) |
|  | Amaurosis | 1 (0.0) | 0 |  | 0 | 0 |
|  | Cataract | 7 (0.3) | 17 (2.4) |  | 1 (0.3) | 7 (1.8) |
|  | Dry eye | 0 | 1 (0.1) |  | 0 | 0 |
|  | Glaucoma | 14 (0.6) | 2 (0.3) |  | 1 (0.3) | 1 (0.3) |
|  | Retinal degeneration | 1 (0.0) | 0 |  | 1 (0.3) | 0 |
|  | Retinal detachment | 3 (0.1) | 0 |  | 0 | 0 |
|  | Retinal haemorrhage | 1 (0.0) | 0 |  | 0 | 0 |
|  | Macular hole | 1 (0.0) | 0 |  | 0 | 0 |
| Gastrointestinal disorders, n (%) |  | 159 (6.3) | 49 (7.0) |  | 33 (8.3) | 33 (8.3) |
|  | Chronic gastritis | 4 (0.2) | 1 (0.1) |  | 1 (0.3) | 1 (0.3) |
|  | Colitis | 2 (0.1) | 0 |  | 2 (0.5) | 0 |
|  | Colitis ulcerative | 1 (0.0) | 0 |  | 0 | 0 |
|  | Constipation | 10 (0.4) | 2 (0.3) |  | 2 (0.5) | 0 |
|  | Diverticulum intestinal haemorrhagic | 0 | 1 (0.1) |  | 0 | 1 (0.3) |
|  | Duodenal ulcer | 20 (0.8) | 5 (0.7) |  | 6 (1.5) | 5 (1.3) |
|  | Enterocele | 1 (0.0) | 0 |  | 1 (0.3) | 0 |
|  | Gastric polyps | 1 (0.0) | 2 (0.3) |  | 0 | 1 (0.3) |
|  | Gastric ulcer | 45 (1.8) | 11 (1.6) |  | 10 (2.5) | 8 (2.0) |
|  | Gastric ulcer haemorrhage | 2 (0.1) | 1 (0.1) |  | 0 | 1 (0.3) |
|  | Gastritis | 5 (0.2) | 3 (0.4) |  | 1 (0.3) | 2 (0.5) |
|  | Gastrooesophageal reflux disease | 4 (0.2) | 2 (0.3) |  | 0 | 1 (0.3) |
|  | Gastroduodenal ulcer | 1 (0.0) | 0 |  | 0 | 0 |
|  | Gastrointestinal haemorrhage | 1 (0.0) | 1 (0.1) |  | 1 (0.3) | 1 (0.3) |
|  | Gastrointestinal necrosis | 1 (0.0) | 0 |  | 0 | 0 |
|  | Gastrointestinal perforation | 2 (0.1) | 0 |  | 1 (0.3) | 0 |
|  | Haemorrhoids | 9 (0.4) | 4 (0.6) |  | 3 (0.8) | 3 (0.8) |
|  | Ileus | 5 (0.2) | 1 (0.1) |  | 0 | 0 |
|  | Inguinal hernia | 8 (0.3) | 4 (0.6) |  | 3 (0.8) | 1 (0.3) |
|  | Intestinal obstruction | 5 (0.2) | 5 (0.7) |  | 0 | 3 (0.8) |
|  | Lumbar hernia | 1 (0.0) | 0 |  | 1 (0.3) | 0 |
|  | Pancreatitis acute | 2 (0.1) | 0 |  | 0 | 0 |
|  | Pancreatitis chronic | 1 (0.0) | 0 |  | 0 | 0 |
|  | Peptic ulcer | 1 (0.0) | 0 |  | 0 | 0 |
|  | Rectal polyp | 1 (0.0) | 0 |  | 0 | 0 |
|  | Salivary gland calculus | 1 (0.0) | 0 |  | 0 | 0 |
|  | Upper gastrointestinal haemorrhage | 1 (0.0) | 0 |  | 0 | 0 |
|  | Vomiting | 0 | 1 (0.1) |  | 0 | 1 (0.3) |
|  | Large intestine polyp | 24 (0.9) | 4 (0.6) |  | 1 (0.3) | 4 (1.0) |
|  | Varices oesophageal | 0 | 1 (0.1) |  | 0 | 0 |
| General disorders and administration site conditions, n (%) |  | 7 (0.3) | 1 (0.1) |  | 1 (0.3) | 1 (0.3) |
|  | Hernia | 2 (0.1) | 1 (0.1) |  | 0 | 1 (0.3) |
|  | Injection site necrosis | 1 (0.0) | 0 |  | 0 | 0 |
|  | Pain | 2 (0.1) | 0 |  | 1 (0.3) | 0 |
|  | Oedema due to renal disease | 1 (0.0) | 0 |  | 0 | 0 |
|  | Adverse drug reaction | 1 (0.0) | 0 |  | 0 | 0 |
| Hepatobiliary disorders, n (%) |  | 71 (2.8) | 25 (3.6) |  | 9 (2.3) | 14 (3.5) |
|  | Alcoholic liver disease | 4 (0.2) | 1 (0.1) |  | 0 | 0 |
|  | Autoimmune hepatitis | 2 (0.1) | 0 |  | 0 | 0 |
|  | Bile duct stone | 1 (0.0) | 2 (0.3) |  | 0 | 1 (0.3) |
|  | Cholecystitis | 3 (0.1) | 3 (0.4) |  | 0 | 3 (0.8) |
|  | Cholecystitis acute | 0 | 1 (0.1) |  | 0 | 1 (0.3) |
|  | Cholelithiasis | 27 (1.1) | 14 (2.0) |  | 5 (1.3) | 7 (1.8) |
|  | Chronic hepatitis | 2 (0.1) | 0 |  | 0 | 0 |
|  | Cirrhosis alcoholic | 2 (0.1) | 0 |  | 0 | 0 |
|  | Hepatic cirrhosis | 4 (0.2) | 2 (0.3) |  | 1 (0.3) | 1 (0.3) |
|  | Hepatic function abnormal | 7 (0.3) | 1 (0.1) |  | 0 | 0 |
|  | Hepatic steatosis | 10 (0.4) | 0 |  | 2 (0.5) | 0 |
|  | Hepatitis | 3 (0.1) | 0 |  | 1 (0.3) | 0 |
|  | Hepatitis acute | 2 (0.1) | 0 |  | 0 | 0 |
|  | Hepatitis alcoholic | 2 (0.1) | 0 |  | 0 | 0 |
|  | Liver disorder | 1 (0.0) | 0 |  | 0 | 0 |
|  | Gallbladder polyp | 1 (0.0) | 1 (0.1) |  | 0 | 1 (0.3) |
| Immune system disorders, n (%) |  | 3 (0.1) | 1 (0.1) |  | 0 | 1 (0.3) |
|  | Atopy | 1 (0.0) | 0 |  | 0 | 0 |
|  | Sarcoidosis | 1 (0.0) | 0 |  | 0 | 0 |
|  | Allergic granulomatous angiitis | 1 (0.0) | 0 |  | 0 | 0 |
|  | Seasonal allergy | 0 | 1 (0.1) |  | 0 | 1 (0.3) |
| Infections and infestations, n (%) |  | 175 (6.9) | 62 (8.8) |  | 27 (6.8) | 42 (10.6) |
|  | Appendicitis | 72 (2.8) | 23 (3.3) |  | 7 (1.8) | 14 (3.5) |
|  | Cellulitis | 1 (0.0) | 0 |  | 0 | 0 |
|  | Chronic hepatitis B | 3 (0.1) | 0 |  | 0 | 0 |
|  | Chronic hepatitis C | 3 (0.1) | 1 (0.1) |  | 0 | 0 |
|  | Chronic sinusitis | 3 (0.1) | 2 (0.3) |  | 0 | 1 (0.3) |
|  | Dermatophytosis of nail | 1 (0.0) | 0 |  | 0 | 0 |
|  | Empyema | 0 | 1 (0.1) |  | 0 | 1 (0.3) |
|  | Epstein-Barr virus infection | 0 | 1 (0.1) |  | 0 | 1 (0.3) |
|  | Gastroenteritis staphylococcal | 1 (0.0) | 0 |  | 0 | 0 |
|  | Hepatitis A | 1 (0.0) | 0 |  | 0 | 0 |
|  | Hepatitis B | 3 (0.1) | 1 (0.1) |  | 0 | 1 (0.3) |
|  | Hepatitis C | 10 (0.4) | 6 (0.9) |  | 6 (1.5) | 2 (0.5) |
|  | Herpes zoster | 4 (0.2) | 0 |  | 0 | 0 |
|  | Lymph node tuberculosis | 0 | 1 (0.1) |  | 0 | 1 (0.3) |
|  | Mastitis | 1 (0.0) | 0 |  | 0 | 0 |
|  | Meningitis viral | 1 (0.0) | 0 |  | 0 | 0 |
|  | Nasopharyngitis | 1 (0.0) | 0 |  | 0 | 0 |
|  | Neurosyphilis | 1 (0.0) | 0 |  | 0 | 0 |
|  | Osteomyelitis | 2 (0.1) | 0 |  | 0 | 0 |
|  | Otitis media | 2 (0.1) | 0 |  | 1 (0.3) | 0 |
|  | Otitis media chronic | 1 (0.0) | 0 |  | 0 | 0 |
|  | Parotitis | 1 (0.0) | 0 |  | 0 | 0 |
|  | Peritonitis | 2 (0.1) | 1 (0.1) |  | 0 | 1 (0.3) |
|  | Pertussis | 1 (0.0) | 0 |  | 0 | 0 |
|  | Pneumonia | 6 (0.2) | 0 |  | 2 (0.5) | 0 |
|  | Pneumonia mycoplasmal | 1 (0.0) | 0 |  | 0 | 0 |
|  | Poliomyelitis | 2 (0.1) | 0 |  | 0 | 0 |
|  | Pulmonary mycosis | 1 (0.0) | 0 |  | 0 | 0 |
|  | Pulmonary tuberculosis | 27 (1.1) | 12 (1.7) |  | 7 (1.8) | 9 (2.3) |
|  | Pyelonephritis | 4 (0.2) | 0 |  | 1 (0.3) | 0 |
|  | Pyonephrosis | 1 (0.0) | 0 |  | 0 | 0 |
|  | Renal tuberculosis | 1 (0.0) | 1 (0.1) |  | 0 | 1 (0.3) |
|  | Rhinitis | 1 (0.0) | 0 |  | 0 | 0 |
|  | Sepsis | 0 | 1 (0.1) |  | 0 | 1 (0.3) |
|  | Sinusitis | 2 (0.1) | 1 (0.1) |  | 0 | 1 (0.3) |
|  | Tuberculosis | 5 (0.2) | 4 (0.6) |  | 1 (0.3) | 4 (1.0) |
|  | Tuberculous pleurisy | 0 | 1 (0.1) |  | 0 | 0 |
|  | Viral hepatitis carrier | 3 (0.1) | 0 |  | 1 (0.3) | 0 |
|  | Anal abscess | 1 (0.0) | 0 |  | 0 | 0 |
|  | Abdominal abscess | 1 (0.0) | 0 |  | 0 | 0 |
|  | Lung infection | 1 (0.0) | 0 |  | 1 (0.3) | 0 |
|  | Atypical mycobacterial infection | 0 | 1 (0.1) |  | 0 | 1 (0.3) |
|  | Syphilis | 0 | 1 (0.1) |  | 0 | 1 (0.3) |
|  | Herpes zoster oticus | 0 | 1 (0.1) |  | 0 | 1 (0.3) |
|  | Otitis media bacterial | 1 (0.0) | 0 |  | 0 | 0 |
|  | Infectious pleural effusion | 1 (0.0) | 1 (0.1) |  | 0 | 0 |
|  | Pneumocystis jirovecii pneumonia | 0 | 1 (0.1) |  | 0 | 1 (0.3) |
|  | Aspergillus infection | 1 (0.0) | 0 |  | 0 | 0 |
| Injury, poisoning and procedural complications, n (%) |  | 29 (1.1) | 13 (1.8) |  | 5 (1.3) | 9 (2.3) |
|  | Alcohol poisoning | 0 | 1 (0.1) |  | 0 | 1 (0.3) |
|  | Ankle fracture | 1 (0.0) | 0 |  | 0 | 0 |
|  | Clavicle fracture | 3 (0.1) | 0 |  | 0 | 0 |
|  | Complications of transplanted kidney | 1 (0.0) | 0 |  | 1 (0.3) | 0 |
|  | Femur fracture | 5 (0.2) | 1 (0.1) |  | 0 | 0 |
|  | Fracture | 1 (0.0) | 0 |  | 1 (0.3) | 0 |
|  | Fractured ischium | 1 (0.0) | 0 |  | 0 | 0 |
|  | Hand fracture | 0 | 1 (0.1) |  | 0 | 1 (0.3) |
|  | Humerus fracture | 1 (0.0) | 0 |  | 0 | 0 |
|  | Injury | 3 (0.1) | 1 (0.1) |  | 1 (0.3) | 0 |
|  | Jaw fracture | 1 (0.0) | 0 |  | 0 | 0 |
|  | Rib fracture | 0 | 1 (0.1) |  | 0 | 1 (0.3) |
|  | Road traffic accident | 1 (0.0) | 0 |  | 0 | 0 |
|  | Spinal compression fracture | 0 | 2 (0.3) |  | 0 | 2 (0.5) |
|  | Subdural haematoma | 4 (0.2) | 0 |  | 1 (0.3) | 0 |
|  | Tendon rupture | 0 | 1 (0.1) |  | 0 | 1 (0.3) |
|  | Tooth injury | 1 (0.0) | 0 |  | 0 | 0 |
|  | Ulna fracture | 0 | 1 (0.1) |  | 0 | 1 (0.3) |
|  | Wrist fracture | 2 (0.1) | 1 (0.1) |  | 0 | 0 |
|  | Vascular pseudoaneurysm | 1 (0.0) | 0 |  | 1 (0.3) | 0 |
|  | Lumbar vertebral fracture | 0 | 1 (0.1) |  | 0 | 0 |
|  | Thermal burn | 1 (0.0) | 0 |  | 0 | 0 |
|  | Pelvic fracture | 1 (0.0) | 0 |  | 0 | 0 |
|  | Gastrointestinal injury | 1 (0.0) | 0 |  | 0 | 0 |
|  | Lower limb fracture | 0 | 1 (0.1) |  | 0 | 1 (0.3) |
|  | Neck injury | 0 | 1 (0.1) |  | 0 | 1 (0.3) |
| Investigations, n (%) |  | 9 (0.4) | 3 (0.4) |  | 7 (1.8) | 1 (0.3) |
|  | Amylase increased | 1 (0.0) | 0 |  | 1 (0.3) | 0 |
|  | Blood cholesterol increased | 1 (0.0) | 0 |  | 1 (0.3) | 0 |
|  | Blood triglycerides increased | 1 (0.0) | 0 |  | 1 (0.3) | 0 |
|  | Blood urea | 0 | 1 (0.1) |  | 0 | 0 |
|  | Cardiac murmur | 0 | 1 (0.1) |  | 0 | 0 |
|  | Gamma-glutamyltransferase increased | 2 (0.1) | 0 |  | 2 (0.5) | 0 |
|  | Lipase increased | 1 (0.0) | 0 |  | 1 (0.3) | 0 |
|  | Platelet count increased | 1 (0.0) | 0 |  | 0 | 0 |
|  | Blood alkaline phosphatase increased | 1 (0.0) | 0 |  | 1 (0.3) | 0 |
|  | Hepatitis C virus test positive | 0 | 1 (0.1) |  | 0 | 1 (0.3) |
|  | Hepatitis B surface antibody positive | 1 (0.0) | 0 |  | 0 | 0 |
| Metabolism and nutrition disorders, n (%) |  | 181 (7.1) | 63 (9.0) |  | 24 (6.0) | 29 (7.3) |
|  | Diabetes mellitus | 114 (4.5) | 36 (5.1) |  | 14 (3.5) | 15 (3.8) |
|  | Gout | 9 (0.4) | 3 (0.4) |  | 2 (0.5) | 0 |
|  | Hypercholesterolaemia | 3 (0.1) | 0 |  | 0 | 0 |
|  | Hypertriglyceridaemia | 1 (0.0) | 0 |  | 0 | 0 |
|  | Hyperuricaemia | 24 (0.9) | 9 (1.3) |  | 2 (0.5) | 7 (1.8) |
|  | Hyperlipidaemia | 30 (1.2) | 14 (2.0) |  | 6 (1.5) | 7 (1.8) |
|  | Type 2 diabetes mellitus | 0 | 1 (0.1) |  | 0 | 0 |
| Musculoskeletal and connective tissue disorders, n (%) |  | 46 (1.8) | 20 (2.8) |  | 13 (3.3) | 12 (3.0) |
|  | Arthralgia | 1 (0.0) | 0 |  | 0 | 0 |
|  | Arthritis | 1 (0.0) | 0 |  | 1 (0.3) | 0 |
|  | Back pain | 1 (0.0) | 1 (0.1) |  | 0 | 1 (0.3) |
|  | Cervical spinal stenosis | 0 | 1 (0.1) |  | 0 | 1 (0.3) |
|  | Lumbar spinal stenosis | 1 (0.0) | 1 (0.1) |  | 0 | 1 (0.3) |
|  | Musculoskeletal pain | 1 (0.0) | 0 |  | 0 | 0 |
|  | Osteoarthritis | 5 (0.2) | 4 (0.6) |  | 1 (0.3) | 2 (0.5) |
|  | Osteoporosis | 3 (0.1) | 1 (0.1) |  | 0 | 0 |
|  | Rhabdomyolysis | 0 | 1 (0.1) |  | 0 | 1 (0.3) |
|  | Rheumatoid arthritis | 5 (0.2) | 1 (0.1) |  | 3 (0.8) | 0 |
|  | Scoliosis | 2 (0.1) | 0 |  | 1 (0.3) | 0 |
|  | Spinal column stenosis | 7 (0.3) | 4 (0.6) |  | 2 (0.5) | 2 (0.5) |
|  | Spinal osteoarthritis | 2 (0.1) | 1 (0.1) |  | 0 | 1 (0.3) |
|  | Neck mass | 0 | 1 (0.1) |  | 0 | 0 |
|  | Intervertebral disc protrusion | 15 (0.6) | 2 (0.3) |  | 4 (1.0) | 2 (0.5) |
|  | Spinal ligament ossification | 0 | 1 (0.1) |  | 0 | 0 |
|  | Intervertebral disc disorder | 0 | 1 (0.1) |  | 0 | 1 (0.3) |
|  | Rheumatic disorder | 2 (0.1) | 0 |  | 1 (0.3) | 0 |
| Neoplasms benign, malignant and unspecified (incl cysts and polyps), n (%) |  | 243 (9.6) | 105 (14.9) |  | 42 (10.6) | 53 (13.4) |
|  | Acoustic neuroma | 1 (0.0) | 0 |  | 0 | 0 |
|  | Acute myeloid leukaemia | 1 (0.0) | 0 |  | 0 | 0 |
|  | Benign mediastinal neoplasm | 0 | 1 (0.1) |  | 0 | 0 |
|  | Benign neoplasm of islets of Langerhans | 1 (0.0) | 0 |  | 1 (0.3) | 0 |
|  | Benign neoplasm of thyroid gland | 1 (0.0) | 0 |  | 0 | 0 |
|  | Bladder cancer | 7 (0.3) | 9 (1.3) |  | 1 (0.3) | 3 (0.8) |
|  | Bladder neoplasm | 1 (0.0) | 0 |  | 0 | 0 |
|  | Bone neoplasm | 1 (0.0) | 0 |  | 1 (0.3) | 0 |
|  | Breast cancer | 13 (0.5) | 5 (0.7) |  | 3 (0.8) | 2 (0.5) |
|  | Carcinoid tumour pulmonary | 1 (0.0) | 0 |  | 0 | 0 |
|  | Cervix carcinoma | 2 (0.1) | 0 |  | 0 | 0 |
|  | Chronic lymphocytic leukaemia | 1 (0.0) | 0 |  | 0 | 0 |
|  | Chronic myeloid leukaemia | 1 (0.0) | 0 |  | 0 | 0 |
|  | Colon cancer | 21 (0.8) | 16 (2.3) |  | 1 (0.3) | 9 (2.3) |
|  | Gallbladder cancer | 1 (0.0) | 0 |  | 0 | 0 |
|  | Gastric cancer | 20 (0.8) | 17 (2.4) |  | 5 (1.3) | 13 (3.3) |
|  | Haemangioblastoma | 2 (0.1) | 0 |  | 0 | 0 |
|  | Haemangioma | 1 (0.0) | 0 |  | 0 | 0 |
|  | Haemangioma of liver | 1 (0.0) | 0 |  | 0 | 0 |
|  | Laryngeal cancer | 4 (0.2) | 1 (0.1) |  | 1 (0.3) | 1 (0.3) |
|  | Lipoma | 1 (0.0) | 1 (0.1) |  | 0 | 1 (0.3) |
|  | Lymphoma | 1 (0.0) | 0 |  | 1 (0.3) | 0 |
|  | Malignant melanoma | 1 (0.0) | 0 |  | 0 | 0 |
|  | Malignant neoplasm of spinal cord | 1 (0.0) | 0 |  | 0 | 0 |
|  | Maxillofacial sinus neoplasm | 1 (0.0) | 0 |  | 0 | 0 |
|  | Mediastinum neoplasm | 2 (0.1) | 0 |  | 1 (0.3) | 0 |
|  | Meningioma | 2 (0.1) | 0 |  | 0 | 0 |
|  | Metastases to adrenals | 2 (0.1) | 0 |  | 0 | 0 |
|  | Metastases to bone | 2 (0.1) | 0 |  | 0 | 0 |
|  | Metastases to liver | 1 (0.0) | 1 (0.1) |  | 0 | 0 |
|  | Metastases to lung | 1 (0.0) | 0 |  | 0 | 0 |
|  | Neoplasm malignant | 0 | 1 (0.1) |  | 0 | 0 |
|  | Non-Hodgkin's lymphoma | 0 | 1 (0.1) |  | 0 | 0 |
|  | Ovarian cancer | 2 (0.1) | 0 |  | 1 (0.3) | 0 |
|  | Pancreatic carcinoma | 5 (0.2) | 0 |  | 0 | 0 |
|  | Parathyroid tumour benign | 0 | 1 (0.1) |  | 0 | 1 (0.3) |
|  | Plasma cell myeloma | 1 (0.0) | 0 |  | 0 | 0 |
|  | Rectal cancer | 2 (0.1) | 1 (0.1) |  | 1 (0.3) | 1 (0.3) |
|  | Renal cancer | 21 (0.8) | 13 (1.8) |  | 4 (1.0) | 7 (1.8) |
|  | Rhabdomyoma | 1 (0.0) | 0 |  | 0 | 0 |
|  | Skin cancer | 1 (0.0) | 0 |  | 0 | 0 |
|  | Thyroid neoplasm | 2 (0.1) | 0 |  | 0 | 0 |
|  | Transitional cell cancer of the renal pelvis and ureter | 0 | 1 (0.1) |  | 0 | 0 |
|  | Tumour embolism | 1 (0.0) | 0 |  | 0 | 0 |
|  | Uterine cancer | 2 (0.1) | 1 (0.1) |  | 1 (0.3) | 1 (0.3) |
|  | Uterine leiomyoma | 34 (1.3) | 9 (1.3) |  | 9 (2.3) | 3 (0.8) |
|  | Metastases to pancreas | 0 | 1 (0.1) |  | 0 | 1 (0.3) |
|  | Lung cancer metastatic | 2 (0.1) | 0 |  | 0 | 0 |
|  | Gastrointestinal stromal tumour | 1 (0.0) | 0 |  | 0 | 0 |
|  | Metastases to peritoneum | 1 (0.0) | 0 |  | 1 (0.3) | 0 |
|  | Angiomyolipoma | 1 (0.0) | 0 |  | 1 (0.3) | 0 |
|  | Adenocarcinoma pancreas | 1 (0.0) | 0 |  | 0 | 0 |
|  | Splenic neoplasm malignancy unspecified | 1 (0.0) | 0 |  | 0 | 0 |
|  | Brain cancer metastatic | 2 (0.1) | 1 (0.1) |  | 1 (0.3) | 0 |
|  | Hepatic cancer metastatic | 4 (0.2) | 1 (0.1) |  | 1 (0.3) | 1 (0.3) |
|  | Retroperitoneal neoplasm | 1 (0.0) | 0 |  | 1 (0.3) | 0 |
|  | Lung neoplasm malignant | 9 (0.4) | 4 (0.6) |  | 1 (0.3) | 2 (0.5) |
|  | Metastases to central nervous system | 2 (0.1) | 0 |  | 0 | 0 |
|  | Prostate cancer | 15 (0.6) | 11 (1.6) |  | 3 (0.8) | 4 (1.0) |
|  | Brain neoplasm | 1 (0.0) | 0 |  | 0 | 0 |
|  | Colon neoplasm | 1 (0.0) | 0 |  | 0 | 0 |
|  | Nasopharyngeal cancer | 1 (0.0) | 0 |  | 0 | 0 |
|  | Testicular neoplasm | 2 (0.1) | 1 (0.1) |  | 1 (0.3) | 1 (0.3) |
|  | Renal neoplasm | 1 (0.0) | 1 (0.1) |  | 0 | 0 |
|  | Salivary gland neoplasm | 1 (0.0) | 0 |  | 0 | 0 |
|  | Lip and/or oral cavity cancer | 0 | 1 (0.1) |  | 0 | 0 |
|  | Ovarian neoplasm | 3 (0.1) | 1 (0.1) |  | 0 | 1 (0.3) |
|  | Adrenal neoplasm | 0 | 1 (0.1) |  | 0 | 1 (0.3) |
|  | Cerebellar tumour | 3 (0.1) | 0 |  | 0 | 0 |
|  | Extranodal marginal zone B-cell lymphoma (MALT type) | 1 (0.0) | 0 |  | 0 | 0 |
|  | Pancreatic neoplasm | 1 (0.0) | 0 |  | 0 | 0 |
|  | Spinal cord neoplasm | 2 (0.1) | 0 |  | 0 | 0 |
|  | Thyroid cancer | 9 (0.4) | 0 |  | 0 | 0 |
|  | Renal cell carcinoma | 4 (0.2) | 1 (0.1) |  | 0 | 0 |
|  | Tumour thrombosis | 0 | 1 (0.1) |  | 0 | 0 |
|  | Hepatic cancer | 2 (0.1) | 0 |  | 0 | 0 |
|  | Hepatic cancer recurrent | 1 (0.0) | 0 |  | 1 (0.3) | 0 |
|  | Hepatocellular carcinoma | 2 (0.1) | 1 (0.1) |  | 0 | 0 |
| Nervous system disorders, n (%) |  | 84 (3.3) | 56 (8.0) |  | 10 (2.5) | 33 (8.3) |
|  | Brain stem infarction | 1 (0.0) | 0 |  | 0 | 0 |
|  | Carotid artery stenosis | 3 (0.1) | 0 |  | 2 (0.5) | 0 |
|  | Carpal tunnel syndrome | 1 (0.0) | 0 |  | 0 | 0 |
|  | Cerebellar haemorrhage | 2 (0.1) | 0 |  | 0 | 0 |
|  | Cerebral haemorrhage | 12 (0.5) | 3 (0.4) |  | 3 (0.8) | 3 (0.8) |
|  | Cerebral infarction | 37 (1.5) | 37 (5.3) |  | 2 (0.5) | 20 (5.0) |
|  | Cerebral thrombosis | 1 (0.0) | 0 |  | 0 | 0 |
|  | Cerebrovascular disorder | 0 | 1 (0.1) |  | 0 | 0 |
|  | Dementia | 0 | 2 (0.3) |  | 0 | 0 |
|  | Dizziness | 4 (0.2) | 0 |  | 0 | 0 |
|  | Epilepsy | 3 (0.1) | 0 |  | 0 | 0 |
|  | Headache | 1 (0.0) | 0 |  | 0 | 0 |
|  | Hydrocephalus | 0 | 1 (0.1) |  | 0 | 0 |
|  | Intracranial aneurysm | 1 (0.0) | 0 |  | 0 | 0 |
|  | Monoplegia | 0 | 1 (0.1) |  | 0 | 0 |
|  | Myelopathy | 0 | 1 (0.1) |  | 0 | 1 (0.3) |
|  | Parkinsonism | 1 (0.0) | 2 (0.3) |  | 0 | 2 (0.5) |
|  | Polyneuropathy | 1 (0.0) | 1 (0.1) |  | 0 | 1 (0.3) |
|  | Sciatica | 1 (0.0) | 1 (0.1) |  | 0 | 1 (0.3) |
|  | Subarachnoid haemorrhage | 5 (0.2) | 0 |  | 1 (0.3) | 0 |
|  | Syncope | 0 | 1 (0.1) |  | 0 | 1 (0.3) |
|  | Transient ischaemic attack | 2 (0.1) | 2 (0.3) |  | 0 | 1 (0.3) |
|  | Vertebral artery stenosis | 1 (0.0) | 0 |  | 0 | 0 |
|  | Vocal cord paralysis | 1 (0.0) | 0 |  | 0 | 0 |
|  | VIIth nerve paralysis | 2 (0.1) | 0 |  | 1 (0.3) | 0 |
|  | Lacunar infarction | 0 | 1 (0.1) |  | 0 | 1 (0.3) |
|  | Subacute myelo-opticoneuropathy | 0 | 1 (0.1) |  | 0 | 1 (0.3) |
|  | Parkinson's disease | 4 (0.2) | 0 |  | 1 (0.3) | 0 |
|  | Basal ganglia haemorrhage | 0 | 1 (0.1) |  | 0 | 1 (0.3) |
| Pregnancy, puerperium and perinatal conditions, n (%) |  | 6 (0.2) | 0 |  | 1 (0.3) | 0 |
|  | Ectopic pregnancy | 5 (0.2) | 0 |  | 1 (0.3) | 0 |
|  | Pre-eclampsia | 1 (0.0) | 0 |  | 0 | 0 |
| Psychiatric disorders, n (%) |  | 31 (1.2) | 3 (0.4) |  | 5 (1.3) | 1 (0.3) |
|  | Alcoholism | 2 (0.1) | 0 |  | 0 | 0 |
|  | Anxiety | 1 (0.0) | 0 |  | 1 (0.3) | 0 |
|  | Depression | 12 (0.5) | 2 (0.3) |  | 3 (0.8) | 0 |
|  | Insomnia | 8 (0.3) | 1 (0.1) |  | 1 (0.3) | 1 (0.3) |
|  | Panic disorder | 1 (0.0) | 0 |  | 0 | 0 |
|  | Schizophrenia | 6 (0.2) | 0 |  | 0 | 0 |
|  | Anxiety disorder | 1 (0.0) | 0 |  | 0 | 0 |
| Renal and urinary disorders, n (%) |  | 93 (3.7) | 27 (3.8) |  | 21 (5.3) | 11 (2.8) |
|  | Calculus ureteric | 10 (0.4) | 2 (0.3) |  | 2 (0.5) | 2 (0.5) |
|  | Calculus urinary | 7 (0.3) | 1 (0.1) |  | 1 (0.3) | 1 (0.3) |
|  | Glomerulonephritis | 1 (0.0) | 1 (0.1) |  | 0 | 0 |
|  | Glomerulonephritis chronic | 4 (0.2) | 0 |  | 1 (0.3) | 0 |
|  | Haematuria | 1 (0.0) | 0 |  | 0 | 0 |
|  | Nephritis | 4 (0.2) | 1 (0.1) |  | 2 (0.5) | 0 |
|  | Nephrolithiasis | 12 (0.5) | 4 (0.6) |  | 4 (1.0) | 2 (0.5) |
|  | Nephropathy | 1 (0.0) | 0 |  | 0 | 0 |
|  | Nephrosclerosis | 2 (0.1) | 0 |  | 1 (0.3) | 0 |
|  | Nephrotic syndrome | 5 (0.2) | 1 (0.1) |  | 1 (0.3) | 0 |
|  | Neurogenic bladder | 1 (0.0) | 0 |  | 0 | 0 |
|  | Pollakiuria | 1 (0.0) | 0 |  | 0 | 0 |
|  | Renal aneurysm | 1 (0.0) | 0 |  | 0 | 0 |
|  | Renal atrophy | 1 (0.0) | 0 |  | 0 | 0 |
|  | Renal cyst | 0 | 1 (0.1) |  | 0 | 0 |
|  | Renal failure | 4 (0.2) | 2 (0.3) |  | 2 (0.5) | 1 (0.3) |
|  | Urethral caruncle | 0 | 1 (0.1) |  | 0 | 1 (0.3) |
|  | Bladder mass | 1 (0.0) | 0 |  | 0 | 0 |
|  | Diabetic nephropathy | 3 (0.1) | 0 |  | 0 | 0 |
|  | Renal impairment | 3 (0.1) | 1 (0.1) |  | 1 (0.3) | 0 |
|  | Chronic kidney disease | 29 (1.1) | 12 (1.7) |  | 6 (1.5) | 4 (1.0) |
|  | Stress urinary incontinence | 1 (0.0) | 0 |  | 0 | 0 |
|  | Acute kidney injury | 1 (0.0) | 0 |  | 0 | 0 |
| Reproductive system and breast disorders, n (%) |  | 28 (1.1) | 21 (3.0) |  | 6 (1.5) | 11 (2.8) |
|  | Benign prostatic hyperplasia | 19 (0.7) | 20 (2.8) |  | 2 (0.5) | 11 (2.8) |
|  | Cystocele | 1 (0.0) | 0 |  | 1 (0.3) | 0 |
|  | Endometriosis | 2 (0.1) | 0 |  | 0 | 0 |
|  | Menopausal symptoms | 1 (0.0) | 0 |  | 1 (0.3) | 0 |
|  | Ovarian cyst | 4 (0.2) | 0 |  | 1 (0.3) | 0 |
|  | Uterine malposition | 0 | 1 (0.1) |  | 0 | 0 |
|  | Uterine prolapse | 1 (0.0) | 0 |  | 1 (0.3) | 0 |
| Respiratory, thoracic and mediastinal disorders, n (%) |  | 46 (1.8) | 24 (3.4) |  | 5 (1.3) | 14 (3.5) |
|  | Asthma | 16 (0.6) | 8 (1.1) |  | 0 | 4 (1.0) |
|  | Bronchiectasis | 2 (0.1) | 0 |  | 0 | 0 |
|  | Bronchitis chronic | 3 (0.1) | 1 (0.1) |  | 0 | 1 (0.3) |
|  | Chronic obstructive pulmonary disease | 1 (0.0) | 1 (0.1) |  | 0 | 1 (0.3) |
|  | Chronic respiratory failure | 0 | 1 (0.1) |  | 0 | 0 |
|  | Emphysema | 2 (0.1) | 3 (0.4) |  | 1 (0.3) | 1 (0.3) |
|  | Epistaxis | 0 | 1 (0.1) |  | 0 | 1 (0.3) |
|  | Interstitial lung disease | 2 (0.1) | 3 (0.4) |  | 0 | 2 (0.5) |
|  | Laryngeal polyp | 0 | 1 (0.1) |  | 0 | 1 (0.3) |
|  | Lung infiltration | 0 | 1 (0.1) |  | 0 | 1 (0.3) |
|  | Pleural effusion | 1 (0.0) | 0 |  | 1 (0.3) | 0 |
|  | Pleurisy | 0 | 1 (0.1) |  | 0 | 0 |
|  | Pneumothorax | 3 (0.1) | 1 (0.1) |  | 1 (0.3) | 1 (0.3) |
|  | Pneumothorax spontaneous | 4 (0.2) | 0 |  | 0 | 0 |
|  | Pulmonary artery stenosis | 1 (0.0) | 0 |  | 0 | 0 |
|  | Pulmonary embolism | 3 (0.1) | 1 (0.1) |  | 1 (0.3) | 0 |
|  | Pulmonary fibrosis | 1 (0.0) | 0 |  | 0 | 0 |
|  | Pulmonary sarcoidosis | 1 (0.0) | 0 |  | 0 | 0 |
|  | Pulmonary thrombosis | 1 (0.0) | 0 |  | 1 (0.3) | 0 |
|  | Sleep apnoea syndrome | 2 (0.1) | 1 (0.1) |  | 0 | 1 (0.3) |
|  | Vocal cord polyp | 1 (0.0) | 0 |  | 0 | 0 |
|  | Infantile asthma | 2 (0.1) | 0 |  | 0 | 0 |
| Skin and subcutaneous tissue disorders, n (%) |  | 8 (0.3) | 1 (0.1) |  | 2 (0.5) | 1 (0.3) |
|  | Dermatitis | 1 (0.0) | 0 |  | 1 (0.3) | 0 |
|  | Dermatomyositis | 1 (0.0) | 0 |  | 1 (0.3) | 0 |
|  | Palmar-plantar erythrodysaesthesia syndrome | 1 (0.0) | 0 |  | 0 | 0 |
|  | Psoriasis | 2 (0.1) | 1 (0.1) |  | 0 | 1 (0.3) |
|  | Pustular psoriasis | 2 (0.1) | 0 |  | 0 | 0 |
|  | Vascular purpura | 1 (0.0) | 0 |  | 0 | 0 |
| Surgical and medical procedures, n (%) |  | 78 (3.1) | 32 (4.6) |  | 11 (2.8) | 14 (3.5) |
|  | Aortic aneurysm repair | 0 | 1 (0.1) |  | 0 | 0 |
|  | Appendicectomy | 10 (0.4) | 0 |  | 3 (0.8) | 0 |
|  | Caesarean section | 1 (0.0) | 0 |  | 0 | 0 |
|  | Cholecystectomy | 3 (0.1) | 1 (0.1) |  | 0 | 1 (0.3) |
|  | Colectomy total | 0 | 1 (0.1) |  | 0 | 0 |
|  | Coronary artery bypass | 0 | 1 (0.1) |  | 0 | 0 |
|  | Craniotomy | 1 (0.0) | 0 |  | 0 | 0 |
|  | Gamma radiation therapy to brain | 1 (0.0) | 0 |  | 0 | 0 |
|  | Haemodialysis | 3 (0.1) | 1 (0.1) |  | 2 (0.5) | 1 (0.3) |
|  | Hip arthroplasty | 2 (0.1) | 1 (0.1) |  | 1 (0.3) | 0 |
|  | Hydronephrosis repair | 0 | 1 (0.1) |  | 0 | 0 |
|  | Hysterectomy | 0 | 1 (0.1) |  | 0 | 1 (0.3) |
|  | Inguinal hernia repair | 2 (0.1) | 1 (0.1) |  | 1 (0.3) | 0 |
|  | Intra-cerebral aneurysm operation | 0 | 2 (0.3) |  | 0 | 2 (0.5) |
|  | Knee arthroplasty | 0 | 1 (0.1) |  | 0 | 0 |
|  | Lipoma excision | 1 (0.0) | 0 |  | 0 | 0 |
|  | Lung lobectomy | 2 (0.1) | 1 (0.1) |  | 0 | 1 (0.3) |
|  | Mitral valve replacement | 1 (0.0) | 0 |  | 0 | 0 |
|  | Myomectomy | 1 (0.0) | 1 (0.1) |  | 1 (0.3) | 1 (0.3) |
|  | Nephrectomy | 11 (0.4) | 2 (0.3) |  | 0 | 0 |
|  | Ovarian cystectomy | 1 (0.0) | 0 |  | 0 | 0 |
|  | Parathyroidectomy | 1 (0.0) | 0 |  | 0 | 0 |
|  | Renal lesion excision | 1 (0.0) | 0 |  | 0 | 0 |
|  | Renal transplant | 3 (0.1) | 0 |  | 0 | 0 |
|  | Renal tumour excision | 1 (0.0) | 0 |  | 0 | 0 |
|  | Small intestinal resection | 1 (0.0) | 0 |  | 1 (0.3) | 0 |
|  | Splenectomy | 2 (0.1) | 0 |  | 0 | 0 |
|  | Urinary cystectomy | 0 | 1 (0.1) |  | 0 | 1 (0.3) |
|  | Urostomy | 0 | 1 (0.1) |  | 0 | 1 (0.3) |
|  | Ventricular septal defect repair | 1 (0.0) | 0 |  | 0 | 0 |
|  | Lymphadenectomy | 1 (0.0) | 0 |  | 0 | 0 |
|  | Glaucoma surgery | 0 | 1 (0.1) |  | 0 | 0 |
|  | Hip surgery | 0 | 1 (0.1) |  | 0 | 1 (0.3) |
|  | Coronary arterial stent insertion | 2 (0.1) | 0 |  | 0 | 0 |
|  | Intestinal polypectomy | 1 (0.0) | 0 |  | 0 | 0 |
|  | Cholelithotomy | 4 (0.2) | 2 (0.3) |  | 0 | 0 |
|  | Epiphyseal surgery | 1 (0.0) | 0 |  | 0 | 0 |
|  | Polypectomy | 0 | 1 (0.1) |  | 0 | 1 (0.3) |
|  | Otorhinolaryngological surgery | 1 (0.0) | 0 |  | 0 | 0 |
|  | Brain tumour operation | 1 (0.0) | 0 |  | 0 | 0 |
|  | Meningioma surgery | 0 | 1 (0.1) |  | 0 | 1 (0.3) |
|  | Meniscus operation | 1 (0.0) | 0 |  | 0 | 0 |
|  | Intervertebral disc operation | 1 (0.0) | 0 |  | 0 | 0 |
|  | Gastric ulcer surgery | 1 (0.0) | 1 (0.1) |  | 0 | 0 |
|  | Choledocholithotomy | 1 (0.0) | 0 |  | 0 | 0 |
|  | Haemorrhoid operation | 2 (0.1) | 0 |  | 1 (0.3) | 0 |
|  | Mass excision | 1 (0.0) | 0 |  | 0 | 0 |
|  | Adrenalectomy | 0 | 1 (0.1) |  | 0 | 0 |
|  | Oophorectomy | 0 | 1 (0.1) |  | 0 | 0 |
|  | Gastrectomy | 1 (0.0) | 0 |  | 0 | 0 |
|  | Tendon operation | 0 | 1 (0.1) |  | 0 | 1 (0.3) |
|  | Thyroid operation | 1 (0.0) | 0 |  | 0 | 0 |
|  | Thyroidectomy | 1 (0.0) | 1 (0.1) |  | 0 | 0 |
|  | Hysteropexy | 0 | 1 (0.1) |  | 0 | 1 (0.3) |
|  | Lung neoplasm surgery | 1 (0.0) | 0 |  | 0 | 0 |
|  | Cataract operation | 2 (0.1) | 0 |  | 1 (0.3) | 0 |
|  | Vocal cord polypectomy | 2 (0.1) | 1 (0.1) |  | 0 | 0 |
|  | Retinopexy | 1 (0.0) | 0 |  | 0 | 0 |
|  | Bladder neck suspension | 1 (0.0) | 0 |  | 0 | 0 |
|  | Breast conserving surgery | 0 | 1 (0.1) |  | 0 | 0 |
| Vascular disorders, n (%) |  | 268 (10.6) | 121 (17.2) |  | 44 (11.1) | 69 (17.4) |
|  | Aneurysm | 1 (0.0) | 1 (0.1) |  | 0 | 1 (0.3) |
|  | Aortic aneurysm | 7 (0.3) | 5 (0.7) |  | 2 (0.5) | 5 (1.3) |
|  | Aortic dissection | 1 (0.0) | 1 (0.1) |  | 1 (0.3) | 1 (0.3) |
|  | Essential hypertension | 0 | 1 (0.1) |  | 0 | 1 (0.3) |
|  | Hypertension | 249 (9.8) | 106 (15.1) |  | 40 (10.1) | 57 (14.4) |
|  | Shock | 1 (0.0) | 0 |  | 0 | 0 |
|  | Takayasu's arteritis | 1 (0.0) | 0 |  | 0 | 0 |
|  | Thrombosis | 1 (0.0) | 0 |  | 0 | 0 |
|  | Varicose vein | 1 (0.0) | 1 (0.1) |  | 0 | 1 (0.3) |
|  | Subclavian artery stenosis | 0 | 1 (0.1) |  | 0 | 0 |
|  | Deep vein thrombosis | 0 | 2 (0.3) |  | 0 | 1 (0.3) |
|  | Peripheral artery aneurysm | 0 | 1 (0.1) |  | 0 | 1 (0.3) |
|  | Aortic rupture | 1 (0.0) | 0 |  | 0 | 0 |
|  | Arterial disorder | 1 (0.0) | 0 |  | 0 | 0 |
|  | Venous thrombosis limb | 1 (0.0) | 0 |  | 0 | 0 |
|  | Peripheral arterial occlusive disease | 3 (0.1) | 1 (0.1) |  | 1 (0.3) | 1 (0.3) |
|  | Arterial occlusive disease | 0 | 1 (0.1) |  | 0 | 0 |
